# Beyond heading time: *FT-*like genes and spike development in cereals

**DOI:** 10.1093/jxb/ery408

**Published:** 2018-12-24

**Authors:** Haiyang Liu, Song Song, Yongzhong Xing

**Affiliations:** National Key Lab of Crop Genetic Improvement, Huazhong Agricultural University, Wuhan, China

**Keywords:** Barley, *Brachypodium distachyon*, fertility, *FLOWERING LOCUS T*-like (*FT*-like), *FLOWERING LOCUS T2*, flowering time, *Ghd* genes, *Photoperiod-1 (Ppd-1)*, spike development, wheat

## Abstract

This article comments on:

**Shaw LM, Lyu B, Turner R, Li C, Chen F, Han X, Fu D, Dubcovsky J.** 2018. *FLOWERING LOCUS T2* regulates spike development and fertility in temperate cereals. Journal of Experimental Botany **70**, 193–204.

**Development of the grain-bearing organ, or spike (inflorescence) is critical to cereal grain development and yield. Shaw *et al.* (2018) found that *FT2*, the closet paralogue of florigen candidate *FT1* in wheat, had a minor effect on heading date in wheat but a significant contribution to spike development, including regulation of the number of spikelets per spike and sterility in *Brachypodium distachyon*, barley and tetraploid wheat. Not only does this increase our understanding of the regulation of flowering time and grain yield control, but suggests *FT2* is a good candidate for breeding high yield cultivars.**

Flowering time is one of the most crucial target traits in crop breeding programmes due to its high correlation with final grain yield. Flowering plants respond to environmental cues to flower at a suitable time to maximize reproductive success, and so by optimizing this process breeders maximize grain yield (Jung and Muller, 2009). It is a highly complex trait that is determined by both environmental and endogenous factors. Integration of various environmental cues triggers the expression of florigen genes in leaves, in turn activating the expression of floral meristem identity genes in the shoot apical meristem and initiation of reproductive growth (Andres and Coupland, 2012).

Over the past two decades, numerous pleiotropic genes that regulate both flowering time and grain yield have been characterized in crops, such as the *Grain number*, *plant height*, *and heading date* (*Ghd*) gene series, *Ghd7*, *Ghd8*, *Ghd7.1* and *Ghd6* in rice and *Photoperiod-1* (*Ppd-H1*) in barley (Yan *et al.*, 2013; Boden *et al.*, 2015; Zhang *et al.*, 2017). Interestingly, all these genes regulate heading time – the number of days from sowing to emergence of the grain-bearing organ – by suppressing the expression of *FLOWERING LOCUS T-*like (*FT-*like) florigen genes and increasing grain yield. The findings of Shaw *et al.* (2018) add important new information to this body of work, helping show how *FT*-like genes regulate grain yield in cereals.

## Diverse functions of *FT*-like genes

The phosphatidylethanolamine binding protein (PEBP) gene family is involved in regulation of flowering time, seed dormancy and panicle/spike development. It can be divided into three subfamilies: *MOTHER OF FT AND TFL1-*like (*MFT-*like), *TERMINAL FLOWER1-*like (*TFL1-*like) and *FT-*like. The *MFT*-like subfamily, the proposed ancestor of the other two subfamilies, regulates seed germination, flowering time and spikelets per panicle. The duplication and diversification of *MFT*-like genes eventually resulted in the *FT*-like and *TFL1*-like subfamilies (Wickland and Hanzawa, 2015). Comparison of the phenotypes donated by *FT-*like and *TFL1-*like genes in cereals (Table 1) shows that *FT*-like genes mainly induce flowering, while *TFL1*-like genes mainly possess anti-florigen activity which represses flowering.

**Table 1.** *FT2* and its homologue(s) in rice, maize, barley and wheat

Species	Clade	Genes	Flowering time	Floret number	Reference
Rice	*FT*-like	*Hd3a*/*OsFTL2*	Earlier flowering	Decrease	Kojima *et al.*, 2002
	*FT*-like	*RFT1*/*OsFTL3*	Earlier flowering	Decrease	Zhao *et al.*, 2015; Zhu *et al.*, 2017
	*FT*-like	*OsFTL1*	Earlier flowering	Decrease	Izawa *et al.*, 2002
	*TFL1*-like	*RCN1-4*	Later flowering	Increase	Kaneko-Suzuki *et al.*, 2018
Maize	*TFL1*-like	*ZCN1*	Later flowering	Increase (in tassel)	Danilevskaya *et al.*, 2010
	*TFL1*-like	*ZCN2*	Much later flowering	Increase (in tassel)	Danilevskaya *et al.*, 2010
	*TFL1*-like	*ZCN3*	Unchanged	Increase (in tassel)	Danilevskaya *et al.*, 2010
	*TFL1*-like	*ZCN4*	Much later flowering	Increase (in tassel)	Danilevskaya *et al.*, 2010
	*TFL1*-like	*ZCN5*	Much later flowering	Increase (in tassel)	Danilevskaya *et al.*, 2010
	*TFL1*-like	*ZCN6*	Unchanged	Increase (in tassel)	Danilevskaya *et al.*, 2010
	*FT*-like	*ZCN8*	Earlier flowering	Decrease	Danilevskaya *et al.*, 2011; Meng *et al.*, 2011
Barley	*FT*-like	*HvFT1*/*VRN3*	Earlier flowering	Unknown	Kikuchi *et al.*, 2009
	*FT*-like	*HvFT2*	Earlier flowering	Unknown	Kikuchi *et al.*, 2009
	*FT*-like	*HvFT3*/*Ppd-H2*	Earlier flowering	Unchanged in LD but aborted in SD	Kikuchi *et al.*, 2009; Mulki *et al.*, 2018
	*TFL1*-like	*HvCEN*	Later flowering	Increased yield	Comadran *et al.*, 2012
Wheat	*FT*-like	*TaFT1*/*VRN3*	Earlier flowering	Decrease	Lv *et al.*, 2014
	*FT*-like	*TaFT2*	Slightly earlier flowering	Increase	Shaw *et al.*, 2018
	*FT*-like	*TaFT3*	Earlier flowering	Unknown	Zikhali *et al.*, 2017

Lv *et al.* (2014) reported *FT1* as a florigen candidate promoting flowering in wheat. Shaw *et al.* (2018) add to this with their characterization of *FT2*, the closest paralogue of *FT1*, in the tetraploid wheat variety Kronos. The *ft2* mutant shows slightly delayed heading time but greatly increased number of spikelets per spike and florets per spikelet. Although *FT2* is highly expressed in leaves and under the control of *VRN1*, *VRN2* and *Ppd-1*, the null mutant only causes a delay in heading time of 2–4 days; this compares with the null mutant of *FT1* which causes a delay of more than 20 days. Therefore, FT2 is not primarily acting as a florigen despite functional *FT2* being expressed in leaves. The double null mutant of *ft1* and *ft2* has a comparable flowering time to the *ft1* single mutant, which indicates that the *FT2* effect is independent of *FT1*, and other florigen genes exist besides *FT1* in wheat.

Unlike other *FT*-like and *TFL1*-like genes, most of which have major effects on heading time, *FT2* shows a major effect on spikelets per spike and fertility but minor effect on heading time. Besides being highly expressed in leaves, *FT2* is also highly expressed in the distal part of the spike. The *ft2* mutant greatly increases the floret number and dramatically reduces the fertility of florets on the spike (Shaw *et al.*, 2018). Thus, *FT2* mainly appears to function in spike development. Notably, sterility is observed not only in the *ft2* mutant in wheat, but also in transgenic knockdown *B. distachyon* and barley, indicating for the first time that *FT2* control of fertility may be a conserved function in temperate cereals. It is clear that *FT*-like subfamily genes also exhibit some degree of sub-functionalization in cereals.

## Spikelets per spike and heading time

Florigen interacts with the bZIP transcription factor FD and 14-3-3 proteins in the shoot apical meristem to form the florigen activation complex. This activates floral meristem identity genes such as *OsMADS15* in rice or *VRN1* in wheat (Li and Dubcovsky, 2008; Taoka *et al.*, 2011), which initiates the transition from the vegetative to the reproductive phase. Next, the shoot apical meristem begins to generate the inflorescence meristem. *RICE CENTRORADIALIS* (*RCN*) belongs to the *TFL1-*like subfamily. RCNs are expressed in the vasculature and move to the shoot apical meristem, where they compete with Hd3a for FD and 14-3-3 to form the florigen repression complex and regulate inflorescence development (Kaneko-Suzuki *et al.*, 2018). Members of the PEBP family seem to interact with FD and 14-3-3 to form a complex. Different complexes and their functions in heading time control and later panicle/spike development are still to be confirmed.

Overexpression of *RCN* greatly delays heading time and generates a larger inflorescence meristem, which produces a long and dense panicle in rice (Kyozuka *et al.*, 2014; Kaneko-Suzuki *et al.*, 2018). The heading date genes upstream of florigen genes such as *Ghd7* in rice probably induce a larger inflorescence meristem by greatly delaying heading date, and finally significantly increase grain yield. Moreover, it seems that plants with a reasonable delay of heading time produce more spikelets per panicle and more grain yield (Zhang *et al.*, 2015).

*FT2* represents another type of *FT*-like genes that greatly increase number of spikelets per spike (spike size) but with minor effects on flowering time. *FT2* is mainly expressed in the inflorescence meristem to increase spikelets per spike after phase transition. This working model is similar to the scenario that *OsMFT1* mainly functions in inflorescence meristem to down-regulate the expression of floral identity genes (e.g. *FZP*, *OsMADS1*) as floral organ determinants (Song *et al.*, 2018). To this end, the spikelets per panicle trait controlled by floral identity genes such as *FZP* is usually independent of flowering time (Bai *et al.*, 2017).

## Potential breeding applications

Negative effects of *FT2* on fertility limits its potential for breeding high yield wheat cultivars without significantly delaying heading date (Shaw *et al.*, 2018). Low fertility of both *FT2* knockout mutants in wheat and RNAi mutants in barley indicates that non-functional or very low expression *FT2* alleles are unlikely to be widely distributed. However, promoter variation could enable a moderate change in *FT2* transcript level. Thus, investigation of natural variation of *FT2* in wheat germplasm should be encouraged to seek regulatory elements which can generate a favourable *FT2* allele to maximize grain yield by balancing the trade-off between spikelets per spike and fertility. This potential *FT2* allele would be more valuable in developing high yield cultivars regardless of *FT1* background. Alternatively, a series of *FT2* RNAi plants with varied interference are worth generating by transformation: the new germplasm with optimized transcript level of *FT2* would be screened in terms of yield performance from these RNAi plants. As suggested by the authors, the *Ft-B1*-Hope allele could probably compensate for the negative effect on fertility of *FT2* (Shaw *et al.*, 2018). Identification of its downstream genes by ChIP sequencing and screening FT2-interaction proteins would help to separate the positive effects on spikelet per spike and negative effects on fertility.

To conclude, the features of *FT2* provide us new opportunities to understand the mechanism of *FT*-like gene in flowering time and grain yield control. If its negative effects on fertility can be resolved, *FT2* is a good candidate for manipulation in breeding high yield cultivars.
